# A Comprehensive Review of Niobium Nanoparticles: Synthesis, Characterization, Applications in Health Sciences, and Future Challenges

**DOI:** 10.3390/nano15020106

**Published:** 2025-01-12

**Authors:** Muhammad Usman Khalid, Austeja Rudokaite, Alessandro Marcio Hakme da Silva, Monika Kirsnyte-Snioke, Arunas Stirke, Wanessa C. M. A. Melo

**Affiliations:** 1 Department of Functional Materials and Electronics, State Research Institute Centre for Physical Sciences and Technology (FTMC), 10257 Vilnius, Lithuania; muhammad.usmanbajwa@ftmc.lt (M.U.K.); austeja.rudokaite@ftmc.lt (A.R.); monika.kirsnyte@ftmc.lt (M.K.-S.); arunas.stirke@ftmc.lt (A.S.); 2 Bioengineering Graduate Program, Scientific and Technological Institute, Brazil University, São Paulo 05508-220, Brazil; alessandro.silva@ub.edu.br

**Keywords:** nanomaterials, niobium, drug delivery, infection treatment, biomedical application

## Abstract

Niobium nanoparticles (NbNPs) have gained attention as promising materials in biomedical applications due to their exceptional biocompatibility, corrosion resistance, and versatility. These nanoparticles offer potential in drug delivery, imaging, and tissue engineering, where their nanoscale properties allow precise interactions with biological systems. Among niobium-based nanomaterials, niobium pentoxide (Nb_2_O_5_) is the most extensively studied due to its chemical stability, bioactivity, and optical properties. Nb_2_O_5_ nanoparticles have shown significant potential in catalysis, biosensing, and photodynamic therapy, as their stability and reactivity make them ideal for functionalization in advanced biomedical applications. Despite these advantages, challenges remain regarding the biodegradability and long-term retention of NbNPs in biological systems. Their accumulation in tissues can lead to risks such as chronic inflammation or toxicity, emphasizing the importance of designing nanoparticles with controlled clearance and biodegradability. Surface modifications, such as coatings with biocompatible polymers, have demonstrated the ability to mitigate these risks while enhancing therapeutic efficacy. This review provides a comprehensive overview of NbNPs, with a focus on Nb_2_O_5_, highlighting their unique properties, current biomedical applications, and limitations. By addressing the remaining challenges, this work aims to guide the development of safer and more effective niobium-based nanomaterials for future medical innovations.

## 1. Introduction

Nanoparticles have revolutionized the field of biomaterials by offering unprecedented control over material properties, enabling advances in medical applications such as drug delivery, imaging, and tissue engineering. Their small size, large surface area-to-volume ratio, and tunable surface chemistry allow precise interactions with biological systems. Among the diverse materials used in nanoparticle formulations, metallic nanoparticles stand out due to their mechanical strength, thermal stability, and versatility in surface modifications. Niobium nanoparticles (NbNPs) have recently emerged as a promising class of metallic nanomaterials, owing to niobium’s inherent biocompatibility, corrosion resistance, and low allergenicity. These properties make niobium nanoparticles highly attractive for biomedical applications, where long-term stability and safety are critical considerations [1,2].

Niobium-based nanoparticles are absorbed because of their potential to integrate seamlessly into various biomedical systems. Niobium is already well established in applications such as orthopedic implants and dental prosthetics, where its corrosion resistance and biocompatibility are widely recognized. Translating these properties to the nanoscale has opened new avenues for using niobium nanoparticles in targeted drug delivery, bioimaging, and tissue scaffolding. The ability of niobium nanoparticles to support surface functionalization also allows them to be tailored for specific applications, such as enhanced cell adhesion, antibacterial coatings, or controlled drug release. Despite their promising characteristics, the field of niobium nanoparticles remains relatively underexplored compared to other metallic nanoparticles like gold or silver, underscoring the need for focused research [3].

A critical area of interest in niobium nanoparticle research is understanding the role of niobium pentoxide (Nb_2_O_5_), a stable oxide form of niobium. Niobium pentoxide is definitely the most studied form of niobium in nanotechnology and biomaterials due to its unique combination of chemical stability, bioactivity, and optical properties. Nb_2_O_5_ nanoparticles have demonstrated potential in catalysis, photonics, and biomedicine, with notable applications in biosensing and drug delivery. Their ability to form a stable oxide layer not only enhances biocompatibility but also provides a reactive surface for functionalization, allowing for diverse biomedical applications. This has made niobium pentoxide a focal point in the broader exploration of niobium-based nanomaterials [4,5].

The potential biomedical applications of niobium pentoxide nanoparticles are further amplified by their photocatalytic and electrical properties. These features make them suitable for advanced applications such as photodynamic therapy and bioelectronics, where their responsiveness to light and electrical stimuli can be leveraged. Research has shown that Nb_2_O_5_ can support cell growth while minimizing adverse effects, making it an ideal candidate for next-generation tissue engineering scaffolds and implantable devices. Furthermore, their stability in biological environments addresses one of the critical challenges faced by metallic nanoparticles: the risk of degradation or toxicity over time. These characteristics position niobium pentoxide nanoparticles as a key player in the development of multifunctional biomaterials [6,7].

Despite these advances, significant knowledge gaps remain in the understanding of niobium pentoxide nanoparticles’ biodegradability, clearance, and long-term biological interactions. While initial studies have demonstrated their safety and efficacy in controlled settings, more research is needed to fully explore their potential and address challenges such as nanoparticle accumulation and long-term toxicity [8]. This review aims to provide a comprehensive overview of niobium nanoparticles, with a particular focus on niobium pentoxide, in the period of 2017 to 2024, highlighting their unique properties, current applications, and future prospective in the field of biomaterials. By consolidating existing knowledge and identifying areas for further investigation, this work seeks to advance the development of niobium-based nanoparticles as versatile and safe tools for biomedical innovation.

## 2. Synthesis and Characterization of Niobium Nanoparticles

### 2.1. Synthesis Methods

**Figure 1:**
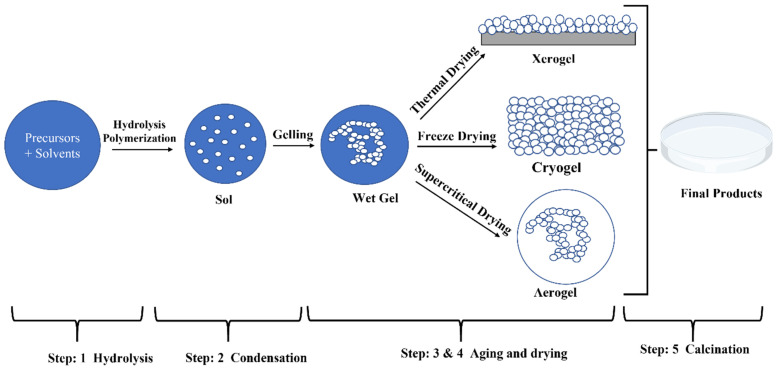
Sol–gel synthesis method.

The sol–gel method is a versatile bottom-up synthesis process that has proven useful in producing various metal oxides, including Nb_2_O_5_ nanoparticles. It is characterized as a “wet chemical” process since it requires a solution phase precursor, in which metal alkoxides are dissolved in an organic solvent to form a stable colloidal suspension, or “sol”. The sol then undergoes hydrolysis and polycondensation processes, resulting in the creation of a “gel” network—a continuous solid structure that contains both solid and liquid phases. This gel can then be dried and calcined to form the required oxide nanoparticles, as shown in Figure 1. The sol–gel method enables the synthesis of nanoparticles with a high degree of purity, fine particle size, controlled porosity, and a large specific surface area, all of which are critical for catalytic and biomedical applications [5,9]. The sol–gel process has a significant advantage in that it can function at very low temperatures, making it energy-efficient and perfect for synthesizing thermally sensitive compounds. Conventional oxide production processes, such as solid-state reactions or chemical vapor deposition, sometimes require temperatures ranging from 400 °C to 3600 °C. In contrast, the sol–gel technique can provide comparable results at temperatures ranging from 70 °C to 320 °C, making it an appealing option for applications with limited thermal budgets [10].

The sol–gel method involves dissolving the molecular precursor in alcohol or water, heating it, and stirring it to cause hydrolysis to turn it into a gel [11]. The acquired damp gel needs to be dried (for example, the alcoholic solution drying process is alcohol burning), powdered, and calcined [12]. Niobium pentoxide (Nb_2_O_5_) can be prepared using metal alkoxide precursors, e.g., niobium (V) ethoxide (Nb (OC_2_H_5_)_5_) with absolute ethanol and ammonia [5,11] or niobium chloride (NbCl_5_) precursor with citric acid (C_6_H_8_O_7_·H_2_O), ethylene glycol (C_2_H_6_O_2_), deionized water (H_2_O), and hydrogen peroxide (H_2_O_2_) [12] (Table 1).

**Figure 2:**
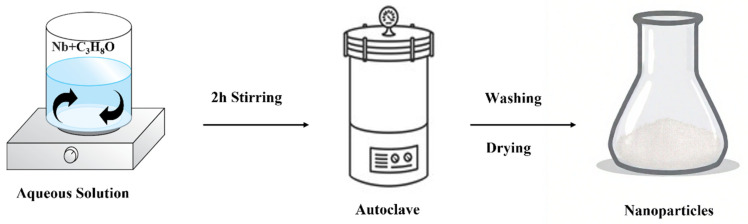
Hydrothermal synthesis method.

Another method for Nb_2_O_5_ nanoparticle production is hydrothermal synthesis, as shown in Figure 2. It is one of the most effective liquid-phase synthesis methods for producing crystallographically and morphologically controlled ceramic oxide nanoparticles [27]. This is because high temperatures and high pressures in an autoclave speed up the hydrolysis of starting materials and the crystallization of ceramic nanoparticles [27]. The advantages of this technique include low-temperature (up to 300 °C) usage, low energy consumption, and inexpensive equipment and precursors—a straight path to fine, homogenous oxide powders [28,29]. Hydrothermal synthesis starts with dissolving niobium oxide in water or other solvents to create a precursor solution and placing it in a vessel under controlled high pressure and heat, creating an environment where niobium ions react to form nanoparticles. After a certain period, the autoclave is cooled and the reaction mixture is washed and dried to remove any residual products, obtaining Nb_2_O_5_ nanoparticle powder [30] (Table 2).

### 2.2. Characterization Techniques

Niobium characterization employs a range of advanced techniques to investigate its structural, morphological, and chemical properties. Transmission Electron Microscopy (TEM) provides high-resolution imaging to analyze crystalline defects and grain boundaries. Scanning Electron Microscopy (SEM) complements this by offering detailed surface morphology and microstructure analysis. X-ray Diffraction (XRD) is utilized to determine phase composition, lattice parameters, and crystallographic orientations. Fourier Transform Infrared Spectroscopy (FTIR) aids in identifying chemical bonding and surface functional groups. Together, these methods offer a comprehensive understanding of niobium’s physical and chemical characteristics, essential for its applications in superconducting materials, alloys, and catalytic systems [55]. Numerous studies have utilized these characterization techniques to reveal critical insights into niobium’s properties and applications. For instance, TEM analysis of niobium films has demonstrated the presence of nanocrystalline structures, which play a pivotal role in enhancing its mechanical and superconducting properties [56]. Biodegradable magnesium (Mg) alloys, such as AZ31B, benefit from enhanced corrosion resistance and biocompatibility when coated with niobium (Nb) and niobium oxide (Nb_2_O_5_) thin films using sputtering techniques. X-ray Diffraction (XRD) confirmed the crystallinity of the coatings, revealing well-defined peaks corresponding to Nb and Nb_2_O_5_, critical for structural integrity. Scanning Electron Microscopy (SEM) showed a uniform, defect-free surface morphology with tightly packed grains, ensuring improved corrosion resistance. These findings underline the importance of XRD and SEM in characterizing and optimizing Nb coatings for biomedical applications, enabling superior performance in simulated body fluid and promoting cell attachment and growth [57].

FTIR spectroscopy has proven particularly valuable in surface modification studies. For example, researchers used FTIR to confirm the successful production of Nb_2_O_5_ thin films, enabling enhanced corrosion resistance and biocompatibility for biomedical implants [58]. These examples demonstrate the utility of combining these techniques to achieve a multidimensional understanding of niobium’s structural, compositional, and functional attributes, guiding its tailored application in diverse fields such as superconductors, medical devices, and advanced alloys.

### 2.3. Properties of Niobium Nanoparticles (Physicochemical)

Niobium pentoxide is a significant research material due to its structural isotropy and unusual physicochemical properties. It has numerous applications in domains like catalysis, energy storage, drug delivery, and optoelectronics. Nb_2_O_5_ has been recognized for its polymorphism, with over 15 different structural phases. The orthorhombic (T-Nb_2_O_5_), pseudo-hexagonal (TT-Nb_2_O_5_), and monoclinic (H-Nb_2_O_5_) phases are commonly investigated and can be synthesized using several processes, including sol–gel processing, chemical vapor deposition, and hydrothermal synthesis [59]. Physical aspects of NbO_5_, especially its electrical features, such as the band gap, are influenced by its structural phase.

The usefulness of NbO_5_ in particular applications can be strongly impacted by changes in the band gap. In their investigation of the photoconductivity of NbO_5_ polymorphs, for instance, Soares et al. found that polycrystalline H-NbO_5_ displays a constant band gap, which helps to maintain stable electronic behavior. Polycrystalline T-NbO_5_, on the other hand, showed two separate photoconductivity peaks at 3.4 eV and 4.7 eV. These peaks, which highlight the special electrical characteristics of T-Nb_2_O_5_ and may be useful for applications needing multi-level excitation, correspond to the basic absorption edge and a secondary transition from the conduction band to a higher energy state, respectively [60].

Niobium pentoxide has impressive optical characteristics that have been widely explored using spectroscopic ellipsometry and spectrophotometry. These experiments show that Nb_2_O_5_ films have a refractive index ranging from 2.0 to 2.3, indicating a strong interaction with light. Furthermore, the thermal treatment and crystallinity of the film have a major influence on its refractive index. As Nb_2_O_5_ films are annealed from room temperature to 700 °C, their crystallinity increases, affecting the refractive index, which drops from around 2.30 to 2.20 as the material’s structure changes [59,61].

Nb_2_O_5_ films have a distinct electrochromic reaction, particularly when doped or intercalated with ions like H⁺ and Li⁺. This intercalation allows for quick and reversible color changes in Nb_2_O_5_, making it an attractive option for smart window technology and display applications. The degree of this coloration effect is extremely adjustable across the UV, visible, and near-IR bands, allowing for fine control over optical transmission. In its quasi-transparent state, Nb_2_O_5_ may attain transmission rates of over 85%. However, with ion intercalation, the transmission rate drops substantially to less than 10%, essentially blocking light. Furthermore, the optical characteristics of Nb_2_O_5_ can cause distinct color appearances based on the film’s crystallinity. Amorphous or partially crystalline films may be blue, whereas more crystalline layers can be brown. Nb_2_O_5_ has tremendous potential in applications such as optical coatings, smart electronics, and energy-efficient window technologies, which require controlled light transmission and color adaption [62,63].

Nb_2_O_5_ has improved thermal diffusivity and conductivity at high temperatures, making it a suitable choice for heat control in microelectronic devices. Nb_2_O_5_’s ability to sustain enhanced thermal characteristics at elevated temperatures makes it well suited for use in high-performance electronics. It contributes to device longevity and efficiency by alleviating thermal stresses and minimizing overheating. Nb_2_O_5_’s thermal characteristics make it a suitable material for applications requiring efficient heat management in tiny electronic devices [63,64].

In today’s electronics, when circuit miniaturization and power increases generate significant heat, efficient thermal management is critical. These systems’ components must have high thermal conductivity to effectively dissipate heat and a low coefficient of thermal expansion to sustain repeated heating and cooling cycles without mechanical deterioration. Thin-film materials show promise in meeting these requirements. A study employing the time domain thermal reflectance (TDTR) method evaluated the thermal characteristics of Nb_2_O_5_ thin films throughout a temperature range of 25 °C to 500 °C. In this technology, a brief heating laser pulse causes a thermal response in the material, with the following cooling behavior caught in the picosecond range, allowing for exact measurement of thermal properties. The thermal diffusivity of Nb_2_O_5_ thin films increased significantly from 0.43 mm^2^/s at ambient temperature to 0.74 mm^2^/s at increasing temperatures. In addition, when the temperature climbed, the films’ thermal conductivity increased from 1.0 W/mK to 2.3 W/mK [64].

Mechanical characteristics are critical for the growth of flexible electronic systems, which require components to withstand repetitive bending and stretching without performance loss. For flexible electronics, a bending or collapsing radius test is obligatory to verify the robustness of materials under continuous flexing and to notice any potential impact on device output. These tests ensure that each layer of a device, particularly thin films and functional layers, can resist mechanical stress without detaching or experiencing material fatigue. Hota et al. conducted a collapsing radius test on a metal–insulator–metal (MIM) capacitor construction with a ~50 nm Nb_2_O_5_ thin film as the insulating layer generated via sputtering. This test indicated that Nb_2_O_5_ films possess exceptional mechanical flexibility and outstanding electrical stability under bending loads, which are linked to the inherent plasticity of niobium and the low-temperature manufacturing of Nb_2_O_5_ [65].

Thus, to demonstrate the efficiency of Nb_2_O_5_ nanoparticles’ properties, Table 3 provides a comparative summary of their properties and applications in relation to other commonly used nanoparticles.

### 2.4. Biocompatibility and Toxicity

#### 2.4.1. In Vitro Studies

When a biomaterial is inserted into the body, its surface comes into contact with the living tissue first. Consequently, the surface characteristics determine how the live tissue will react to the biomaterial at first. Therefore, there has been a rapid expansion of surface modifications. One of the critical surface properties for orthopedic or dental implants, which is capable of being modified, is corrosion resistance, which determines the product’s service life and prevents harmful corrosion products from being released into living organisms. One of the refractory metals, niobium, has been studied, used in implants, and shown good biocompatibility, viable to be used as a biomaterial. Olivares-Navarrete et al. performed in vitro tests (cellular adhesion, proliferation, and viability) of niobium thin-film coatings using human alveolar bone-derived cells in comparison with samples of medical-grade stainless steel and tissue culture plastics. The results showed that niobium-coated surfaces had better biocompatibility than stainless steel [73]. Another example of an in vitro biocompatibility test of a titanium–niobium alloy (S_TiNb) versus commercially pure smooth titanium (S_TiCp), which are both used in dental implants, evaluated cell adhesion, growth morphology, and viability using human osteoblast-like SaOs-2 and monocyte THP-1 cell lines [74].

The results concluded that S_TiCp and S_TiNb alloys had no significant effect on the proliferation and viability of the SaOs-2 cell model; however, the presence of S_TiNb discs significantly reduced the proliferation and viability of THP-1 cells. This suggests a potentially safe and trouble-free use of smooth titanium–niobium alloys in dental implants and a further need for subsequent experiments and in vivo studies for the biocompatibility of titanium alloys [74].

It is also very important to perform toxicity tests for biomedical applications because isolated elements or their compounds could be released from the implant surface due to corrosion or wear of the device. In a recent article, scientists measured not only the biocompatibility and antibacterial effects but also the cytotoxicity of niobium oxynitride coatings [75]. Metallic oxynitride thin films have become more technologically well known, with known increased biocompatibility due to the presence of oxygen in their structure. In this study, the cytotoxic effects of the coatings were analyzed using human fibroblast cells, confirming them as non-toxic and supporting cell attachment and proliferation [76].

In another example, a cytotoxicity test of niobium (V) oxide nanoparticles in Chinese hamster ovary (CHO-K1) cells showed reduced cell toxicity and harmful effects on cell division [76]. Lastly, the micro- and nanoparticle cytotoxicity of calcium silicate-based cement combined with niobium oxide (Nb_2_O_5_) was evaluated using four cell lines—primary cultures (human dental pulp cells (hDPCs) and human dental follicle cells (hDFCs)) and immortalized cultures (human osteoblast-like cells (Saos-2) and mouse periodontal ligament cells (mPDL)) [77]. MTT and Trypan Blue tests were used for cell viability assessment, demonstrating viability in all cell lines [77].

Additionally, studies have explored the functionalization and coating of niobium nanoparticles for various biomedical applications, particularly in drug delivery and tissue engineering. Niobium, due to its excellent biocompatibility, low toxicity, and ability to form stable oxide layers, has become a promising candidate for use in the development of biomaterials. In one study, researchers functionalized niobium nanoparticles with polyethylene glycol (PEG) to enhance their colloidal stability and improve their interaction with biological tissues. This functionalization not only facilitated the controlled release of drugs but also ensured that the nanoparticles remained stable in physiological environments. The PEG coating also reduced potential immunogenic responses, making these nanoparticles suitable for long-term use in drug delivery systems [78].

Another area of investigation has been the use of niobium for enhancing bone regeneration in tissue engineering. A study demonstrated that niobium nanoparticles, when coated with hydroxyapatite (HA), significantly improved osteoconductivity and promoted osteoblast differentiation. The HA coating mimicked the mineral phase of bone, which enhanced the biocompatibility of niobium nanoparticles and encouraged bone tissue formation. The functionalized niobium–HA composite exhibited high stability and bioactivity, showing great promise for bone regeneration applications. This coating also enabled the controlled release of bioactive ions, further supporting the healing process and the growth of new bone tissue [79].

Additionally, niobium-based nanoparticles have been functionalized for use in cancer therapy. In one study, niobium oxide nanoparticles were coated with a targeting ligand—such as folic acid—to bind to cancer cells expressing folate receptors specifically. This functionalization enabled the nanoparticles to selectively deliver chemotherapeutic agents to tumor cells, minimizing side effects to healthy tissues. Furthermore, the surface modification of niobium nanoparticles with targeting molecules, along with their ability to undergo photothermal effects under laser irradiation, has been explored for use in photothermal therapy for cancer. The functionalized niobium nanoparticles effectively absorbed light and converted it into heat, selectively performing ablation of cancer cells. This dual approach—drug delivery and photothermal treatment—demonstrated the versatility of functionalized niobium nanoparticles in cancer therapy [80].

#### 2.4.2. In Vivo Studies

In biomedical applications, in vivo studies are crucial for developing procedures, medical devices, or surgical instruments and addressing shortcomings from in vitro studies, such as evaluating safety, toxicity, and compatibility in complex models. Matsuno et al. evaluated the biocompatibility of niobium, among other refractory metals, when implanted in rats‘ femoral bone marrow and the abdominal region‘s subcutaneous tissue for 2 or 4 weeks [81]. There was no inflammatory response or dissolution. There was also visible healing around the implant insertion position (surrounded by thin fibrous connective tissue), indicating good biocompatibility [81].

**Figure 3:**
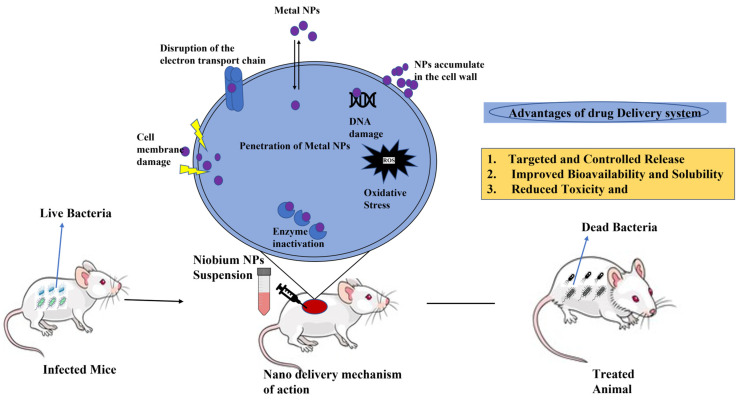
Drug delivery to infected mice.

An in vivo cytotoxicity test was performed on Swiss mice, as shown in Figure 3, after treating them with a single dose of niobium oxide (Nb_2_O_5_) for 3, 7, and 12 days [7]. Data showed cell count increase in the peritoneal area and liver cell regeneration, which indicate cytotoxicity as not being progressive [7].

Recently, researchers administered NbNPs to rats to assess their biodistribution, clearance mechanisms, and potential toxicity [82]. The results revealed that the nanoparticles primarily accumulated in the liver, spleen, and lungs, with minimal retention in other organs. Histopathological analysis indicated mild inflammation and tissue damage in these organs, raising concerns about the long-term retention of NbNPs in vivo. While there was no severe toxicity observed, the study suggested that prolonged exposure could lead to organ-specific stress, particularly oxidative damage in the liver and potential renal tubule degeneration. These findings emphasize the need for careful consideration of dosing regimens and long-term effects when evaluating NbNPs for biomedical use.

Another in vivo study investigated the immunotoxicity of NbNPs by administering them to mice at varying doses [83]. The research focused on the immune responses triggered by the nanoparticles, including cytokine production and immune cell activation. At higher doses, NbNPs induced a significant increase in pro-inflammatory cytokines, suggesting an activation of the immune system. However, lower doses of NbNPs showed minimal immune response, indicating a potential safety margin for controlled exposure. The study concluded that while NbNPs could have immunomodulatory effects, their safety profile could be optimized through careful dose management, highlighting the importance of understanding their impact on immune system function in preclinical evaluations. These in vivo findings provide valuable insight into both the therapeutic potential and risks associated with NbNPs.

A study conducted by Li et al. (2001) [84] explored the long-term effects of NbNPs on liver function in rats, showing that chronic exposure to NbNPs could lead to oxidative stress, which in turn caused cellular damage in liver tissues. The researchers observed an increase in reactive oxygen species (ROS) levels and lipid peroxidation markers, signifying oxidative damage. Histopathological analysis revealed signs of hepatic cell degeneration, though the damage was not as severe as that induced by other types of nanoparticles. The study emphasizes the need for extended studies to assess the long-term liver toxicity of NbNPs, which is critical for evaluating their safety for clinical applications.

Another in vivo study examined the effects of NbNPs on the renal system in rats after both acute and chronic exposure [85]. The results showed that while acute exposure to NbNPs did not induce significant renal damage, prolonged exposure led to renal tubule degeneration and inflammatory cell infiltration in the kidneys. The study suggested that NbNPs may have a dose-dependent nephrotoxic effect, with higher concentrations leading to greater renal impairment. These findings underscore the necessity of considering organ-specific effects, especially the kidneys, when evaluating the safety of NbNPs for medical use.

A separate study focused on the cardiovascular impact of NbNPs in vivo, administering them to rats over several weeks to assess potential vascular toxicity [86]. The results indicated that while there was no significant change in heart function, there were signs of endothelial cell dysfunction in the blood vessels, along with mild arterial thickening. This vascular response suggests that NbNPs could have subtle but important effects on cardiovascular health, particularly with chronic exposure. These findings further emphasize the importance of understanding the full range of potential adverse effects of NbNPs before they can be used in clinical settings, particularly for long-term treatments.

It is crucial to continue in vivo studies, as there is a lack of them, to further elucidate the biocompatibility and cytotoxicity of niobium in living organisms to develop medical solutions.

### 2.5. Biodegradability and Clearance Mechanisms

The biodegradability and clearance mechanisms of NbNPs in the body are essential topics in understanding their biomedical safety and effectiveness. Niobium, a biocompatible metal known for its corrosion resistance, is increasingly being explored for use in nanoparticle form in medical applications like imaging, drug delivery, and tissue engineering. However, the biodegradability of niobium nanoparticles is limited, given niobium’s metallic nature, which raises concerns about long-term retention and possible toxicity if they accumulate in tissues. Studies suggest that surface modifications, such as coating niobium with biodegradable polymers, may enhance their clearance and reduce the potential for persistence in organs, providing a more controlled release and safer profile for in vivo use [87].

The primary clearance mechanisms for niobium nanoparticles largely depend on their size, surface charge, and the specific characteristics of the biological environment. Small niobium nanoparticles, generally below 10 nm, are more likely to pass through renal filtration and be cleared via the urinary system, while larger particles are more likely to be taken up by the mononuclear phagocyte system (MPS), primarily in the liver and spleen [88]. Niobium nanoparticles that evade immediate clearance mechanisms might remain in the body longer, with studies showing that certain surface functionalization can alter the rate of phagocytosis and cellular uptake, particularly by macrophages in the liver and spleen. This accumulation can pose a risk for chronic inflammation or other toxic effects over time, underscoring the importance of designing nanoparticles with a view toward efficient clearance from the body [89].

## 3. Applications of Niobium in Health Sciences

### 3.1. Niobium in Drug Delivery Systems and Therapeutic Applications

Because of its unique physical, optical, and biological properties, such as antibiofilm and antimicrobial ability, niobium can be applied to develop innovative drug delivery systems or act as a part of therapeutic agents. In a recent study, scientists created a delivery platform containing ultrathin niobium carbide (Nb_2_C) nanosheets, which exhibit a large specific surface area, offering plenty of anchoring sites for proteins, drugs, or macromolecules, and berberine (BBR), the plant-derived chemical, showing antibacterial, antioxidative, and anticancer activities, especially metastasis-related protein regulation in the tumor, was integrated into the nanosheets. These nanocomposites were used in combined chemotherapy and photothermal therapy on breast cancer cells, resulting in the efficient elimination of cancer cells and successful metastasis inhibition by regulating the expression of proteins linked with extracellular matrix and epithelial–mesenchymal transition [90]. Studies have also been based on the thermo-plasmonic characteristics of rod-shaped, core–shell, and spherical niobium nanoparticles to evaluate if and which type of niobium nanoparticles can potentially target cancer cells. To investigate these properties, scientists simulated synchrotron radiation emission as a function of the beam energy and niobium nanoparticles and discovered that the laser light stimulates the resonance of surface plasmon of the nanoparticles, converting the absorbed energy into heat which destroys tumor tissue near to nanoparticles without hurting sound tissues [91]. This makes niobium nanoparticles appropriate for tissue, tumor, and optothermal human cancer cell treatment. Another experiment was performed with niobium as a part of a hybrid compound called Wells–Dawson polyoxometalate (POM) covalently bound with folic acid, (Bu_4_N)_5_H_4_[P_2_W_15_Nb_3_O_62_]–folic acid.

In this compound, niobium replaces the tungsten addenda atom, which can increase the oxygen atom’s tendency to form esters when Tris molecules are present, increasing the atom nucleophilicity in the POM structure. The bioactivity of the POM was tested against human fibroblast (HFB), cervical (HeLa), and human breast cancer (MCF-7) cells, showing it effectively, more than 2- to 3-fold more biocompatible and safer than the positive control of methotrexate [92]. The compound successfully knocked down cancer cells with folic acid‘s cytotoxicity due to the overexpression of folate receptors on the cancer cell surface.

A recently published study examined the thermo-plasmonic properties of niobium nanorods for optothermal treatment of human cancer cells, tissues, and tumors under synchrotron radiation. The research demonstrated that niobium nanorods effectively convert absorbed light energy into heat, enabling targeted destruction of cancerous tissues while minimizing damage to surrounding healthy cells. This approach highlights the promising role of niobium nanoparticles in enhancing the efficacy of cancer therapies [93].

The use of niobium carbide (NbC) nanoparticles for photothermal therapy in infected wound treatment has also been reported. The research found that NbC nanoparticles possess excellent near-infrared absorption properties, allowing them to convert laser irradiation into localized heat. This heat effectively eradicates bacteria such as *Staphylococcus aureus* and *Escherichia coli*, promoting faster wound healing. The study underscores the potential of niobium-based nanoparticles in developing advanced antimicrobial therapies [94].

In addition to the studies on cancer therapy and antimicrobial applications, niobium-based materials have also been investigated for their potential in enhancing the bioavailability and controlled release of therapeutic agents. A study reported the use of niobium oxide (Nb_2_O_5_) nanoparticles for the delivery of anticancer drugs. The research highlighted that niobium oxide nanoparticles have high surface area and biocompatibility, which makes them suitable carriers for drug molecules. These nanoparticles were found to release the anticancer drug doxorubicin in a controlled manner, improving the therapeutic index and reducing side effects typically associated with conventional chemotherapy. The study also demonstrated that niobium oxide nanoparticles were able to target tumor cells, leading to an enhanced therapeutic effect compared to traditional drug delivery methods [95].

Moreover, a study focused on the use of niobium as a catalyst for enhancing the therapeutic properties of certain drugs through its effect on cellular uptake mechanisms. The research revealed that niobium-based materials could facilitate the incorporation of therapeutic compounds into cells, promoting better drug penetration. This ability to improve cellular uptake has vast implications for increasing the efficacy of a range of treatments, including those for chronic diseases such as diabetes and cardiovascular conditions. Additionally, the study found that niobium particles exhibited low toxicity and stable biocompatibility, making them ideal candidates for long-term drug delivery applications [96].

In conclusion, niobium is a promising material for applications in the medical field for drug delivery and therapeutics.

### 3.2. Niobium as an Imaging Agent

Mass spectrometry imaging (MSI) is a technology to analyze the spatial distribution of proteins, peptides, metabolites, etc., without chemical labeling, and when applied with enhanced laser desorption/ionization (LDI) performance substrates, it allows the visualization of multiple classes of molecular species. This method can be applied in surgical procedures for precise identification of specific biomarkers, helping to diagnose various diseases, injuries, or other clinical issues. Surface-assisted laser desorption/ionization mass spectrometry imaging (SALDI MSI) using noble-metal nanoparticle matrices is an exceptional biochemical analysis tool [97].

In this particular article, niobium nanoparticles were used as a matrix for SALDI MSI, seeded onto mouse brain slices, leading to enhanced biomolecule fragmentation, making transition-metal (such as niobium) nanoparticles promising linker- and residue-free, cost-effective matrices [97]. Novel MRI-compatible material development is crucial, as MRI has become an essential non-invasive diagnostic tool without X-ray irradiation in modern medicine. Therefore, scientists have developed novel Zr–Mo–Nb alloys for biomedical applications, incorporating niobium because of its high biocompatibility, potential to enhance corrosion resistance, and mechanical performance [5]. Also, mixing niobium (Nb), zirconium (Zr), and molybdenum (Mo) causes an uneven mix of materials, creating a new kind of crystal, called the β-Zr phase, which makes the grain size of the material much smaller. This results in a fine and layered structure of two crystal types (α and β) in the alloy. This is an option for biocompatible, long-lasting, and MRI-compatible materials that can raise the standard of medical care and improve patients’ general well-being [98].

A study explored the use of niobium-based nanoparticles as agents for positron emission tomography (PET) imaging. The research demonstrated that niobium nanoparticles, when labeled with a suitable radioisotope, could effectively target tumor cells, offering high-resolution images for early cancer detection. The study highlighted the excellent biocompatibility of niobium and its promising application for non-invasive diagnostic imaging, suggesting that it could offer a new avenue for visualizing malignant tissue with minimal side effects. Additionally, the use of niobium in PET imaging allowed for the development of tracers with enhanced tissue penetration, which is critical for detecting smaller or deep-seated tumors [99].

In another investigation, niobium nanoparticles were assessed for their potential in magnetic resonance imaging (MRI) as contrast agents. The study revealed that niobium oxide nanoparticles demonstrated significant potential to improve MRI sensitivity due to their unique magnetic properties. These nanoparticles were found to provide a stronger contrast effect in MRI scans compared to conventional agents, particularly in the imaging of vascular tissues and organs like the liver. Niobium’s ability to tune its magnetic properties through surface modification also enables the fine-tuning of imaging contrast, further advancing its utility in precise and high-quality diagnostics. This body of research points toward niobium’s growing role in enhancing the effectiveness of medical imaging techniques, opening doors to more accurate and early detection of various diseases [100].

Additionally, niobium-based materials have been investigated for their role in enhancing the imaging capabilities of other diagnostic techniques, such as X-ray imaging. A study showed the integration of niobium into composite materials for use in X-ray contrast agents. The study found that niobium’s high atomic number contributed to significant attenuation of X-ray beams, providing clearer and sharper images when used as a contrast agent. The niobium-based composite demonstrated improved biocompatibility and stability compared to traditional iodine-based contrast agents. This novel approach to X-ray imaging holds promise for reducing the dose of contrast agents required, potentially minimizing the associated risks while maintaining high-quality diagnostic images. Researchers suggest that niobium’s versatility in medical imaging could extend beyond PET and MRI, providing a broader range of applications in clinical settings [101].

### 3.3. Biosensing

The synthesis of Nb_2_O_5_ nanorod array films and their electrochemical properties are explored for biosensing applications. The unique characteristics of Nb_2_O_5_ nanorods, including high surface area and excellent conductivity, make them ideal for improving the performance of biosensors. Their ability to interact efficiently with biological molecules enhances the sensitivity and accuracy of detection, offering significant potential for the development of advanced biosensors in medical diagnostics, pathogen detection, and real-time monitoring of biological processes [35]. The impact of deposition geometry on the structural, morphological, and optical properties of Nb_2_O_5_ nanostructures prepared via hydrothermal methods was investigated. The study suggests that by carefully controlling deposition parameters, the surface characteristics of Nb_2_O_5_ can be optimized for applications such as biosensing. The enhanced surface area, crystallinity, and optical properties of the nanostructures can improve the efficiency and sensitivity of biosensors, making them more effective for detecting biological molecules and biomarkers [51].

Recently, a study explored the use of niobium-based nanomaterials in electrochemical biosensors for detecting glucose levels in diabetic patients. The researchers modified the niobium surfaces with functionalized nanostructures, enhancing their sensitivity and selectivity towards glucose. The electrochemical response was shown to be significantly improved compared to conventional sensors, providing a more efficient and reliable platform for continuous glucose monitoring. This study highlighted the advantages of niobium’s stable oxide layer, which acts as an ideal surface for immobilizing biomolecules such as enzymes, ensuring high performance even in complex biological environments [102].

In another study, niobium was explored as a key component in the development of a sensor for detecting cancer biomarkers. The research focused on creating a niobium-based field-effect transistor (FET) biosensor, where the surface of the niobium electrode was functionalized with antibodies specific to the biomarker protein. The FET biosensor exhibited a high degree of sensitivity and specificity, capable of detecting trace amounts of the biomarker in serum samples. The study demonstrated niobium’s potential in precision medicine, where early detection of cancer through non-invasive biosensors could lead to improved diagnostic outcomes. Niobium’s robust performance in diverse biological conditions further establishes its promising role in advancing biosensor technologies [103].

Another study focused on the development of a niobium-based surface-enhanced Raman scattering (SERS) biosensor, utilizing niobium’s unique surface properties to amplify Raman signals for highly sensitive biomolecular detection. The researchers incorporated niobium nanoparticles as substrates for the SERS biosensor, demonstrating their ability to enhance the Raman scattering of targeted biomolecules, such as DNA and proteins. The enhanced signal allowed for the detection of low-abundance biomarkers with high specificity, making it a promising approach for early disease diagnosis and environmental monitoring. The study emphasized niobium’s ability to provide stable and reproducible SERS signals, even in complex biological matrices, underscoring its potential in the field of biosensing where high sensitivity and selectivity are crucial [104].

In addition to electrochemical and optical sensing, niobium has also been investigated for its potential in piezoelectric biosensors. A study developed a Nb_2_O_5_ thin-film-based quartz crystal microbalance (QCM) biosensor for the detection of protein interactions. The researchers utilized the high surface area and stability of niobium oxide films to immobilize specific antibodies, enabling the sensitive detection of target proteins in complex biological fluids. The results demonstrated that niobium oxide-based QCM biosensors exhibited excellent sensitivity, with the ability to detect femtomolar concentrations of target proteins. This application highlights niobium’s versatility in biosensing, offering a promising platform for real-time monitoring of protein–protein interactions, which could be crucial for diagnostic and therapeutic purposes in the field of personalized medicine [105].

## 4. Challenges and Future Perspectives

Niobium oxide nanoparticles (Nb_2_O_5_ NPs) have potential in health sciences for medication delivery, imaging, and antibacterial therapies, but confront hurdles in clinical recognition. Among these is the need for a better understanding of their biocompatibility and toxicity; while niobium oxide generally shows positive biocompatibility, nanoparticles can behave unpredictably depending on their size, shape, and surface modifications, occasionally causing oxidative stress or inflammation [106]. Additionally, Nb_2_O_5_ NPs face stability issues, often aggregating in physiological environments, which limits their effectiveness and bioavailability. Complex surface functionalization strategies are necessary to enhance their targeting and reduce toxicity, add to production costs, and raise scalability concerns. Furthermore, limited pharmacokinetic data and a lack of standardized testing protocols complicate regulatory approval, with inconsistencies in synthesis and characterization making it difficult to establish safety benchmarks. Addressing these challenges will require focused research on nanoparticle behavior in biological systems, the development of stable surface modifications, and clear regulatory guidelines, all of which are essential to unlock the full potential of niobium oxide nanoparticles in healthcare.

Niobium oxide nanoparticles have emerged as promising materials in health sciences, with potential applications spanning drug delivery, diagnostic imaging, antimicrobial treatments, and cancer therapy. Future research is anticipated to focus on developing eco-friendly synthesis methods, optimizing surface modifications for targeted delivery, and establishing standardized testing protocols, paving the way for safer and more effective medical applications. As these advancements unfold, niobium-based nanomaterials could become integral components of next-generation therapeutic and diagnostic tools in precision medicine. In this review article, metal oxide niobium was explained with its synthesis methods, characterization techniques, and applications in health sciences (in vitro, in vivo, biocompatibility). In addition, challenges and future perspectives were discussed.

## 5. Author Perspectives

From a biomedical standpoint, NbNPs, particularly Nb_2_O_5_, present a compelling opportunity for advancing therapeutic and diagnostic technologies. The unique properties of NbNPs, such as their excellent biocompatibility, corrosion resistance, and ability to be functionalized for specific applications, make them an attractive choice for a wide range of medical uses, including drug delivery, imaging, and tissue engineering. However, the path to clinical translation requires overcoming critical challenges related to their in vivo behavior. The limited biodegradability of NbNPs and their potential for long-term accumulation necessitate focused research on optimizing surface modifications and bio-coating strategies that can promote faster clearance from the body. Moreover, further investigation into their interaction with immune cells and potential long-term toxicological effects is essential to ensure their safe use in humans.

Looking ahead, the future of niobium-based nanoparticles in nanomedicine lies in their strategic integration with other emerging technologies, such as gene therapy, photothermal treatment, or even regenerative medicine. The potential for NbNPs to target specific tissues or cross biological barriers like the blood–brain barrier could unlock new possibilities for treating complex diseases such as cancer and neurodegenerative disorders. However, a more thorough understanding of their in vivo fate, coupled with advances in manufacturing techniques for producing consistent and scalable niobium nanoparticles, will be key to realizing their full therapeutic potential. The development of standardized synthesis methods and comprehensive safety evaluations will be pivotal in advancing NbNPs from the laboratory to clinical applications, ultimately making them an integral part of the next generation of biomedical nanomaterials.

## Figures and Tables

**Table 1:** A summary of citations followed by sol–gel methods.

Solution	Time (h)	Temp. °C	Morphology	Crystal Phase	Dimensions (nm)	Applications
Niobium alkoxides (Nb(OR)_5_) with acetic acid [13]	4	80	Xerogels and powders	Monolithic, tetragonal when heated above 500 °C	N/A	Nb_2_O_5_ powders were synthesized
Niobium oxide (Nb_x_) with ethanol and HCL [14]	24	25	Xerogels and powders	Amorphous	N/A	Electrochromic material
NbCl_5_ with propanol and acetic acid [15]	1	300–500	Xerogel films	Amorphous at 300 °C and pseudo-hexagonal at 500 °C	N/A	Electrochemical reversibility and dip coating
NbCl_5_ with ethanol and acetic acid [16]	2	450	Thin Films	Amorphous	T: 83–178	Counter electrode, particularly in nickel oxide devices.
NbCl_5_ with ethanol [17]	24	25	Silica aerogel	Amorphous	N/A	N/A
Niobium chloride, NbCl_5_, with tetraethoxysilane [18]	48	25	Dried gels	Amorphous	N/A	N/A
NbCl_5_ with citric acid, ethylene glycol, deionized water, hydrogen peroxide [11]	12	25	Transparent and yellow gel	Below 600 °C, amorphous Orthorhombic when calcined at 900 °C	T: 50, when heated T: >200 nm	Capacitors
Niobium ethoxide with acetic acid [19]	24	300	Thin Films	Tetragonal	N/A	Photocatalytic activity
Anhydrous NbCl_5_ with InCl_3_ and acetylacetone [20]	1	85	Thin Films	Cubic bixbyite	T: 15–25	Highest optical transmittance, conductive films
Niobium ethoxide with tetra-ethyl-ortho-titanate and ethanol [21]	1	450	Thin Films	Amorphous to crystalline	T: 100–370	CO sensing applications
Niobium ethoxide with NH_4_OH [22]	2	25	Nanoparticles	Orthorhombic and hexagonal	D: 1–100	N/A
Niobium chloride (NbCl_5_) with aluminum chloride hexahydrate [23]	1	70	Nanorods and thin films	Hexagonal	N/A	Photocatalytic Activities
Niobium chloride (NbCl_5_) with hydrogen peroxide and coconut water powder [24]	136	25	Powder	Orthorhombic and monoclinic when heated at 1000 °C	N/A	Dielectric constant
Niobium ethoxide with isopropyl alcohol [25]	2	180	Thin Films	Polycrystalline and amorphous structure	N/A	Fabrication of unipolarswitching memory devices
Nb_2_O_5_ powder with hydrofluoric acid [26]	2	100	Nanoparticles	Polycrystalline	N/A	N/A

**Table 2:** A summary of citations followed by hydrothermal synthesis methods.

Solution	Time (h) and Tem. °C	Morphology	Crystal Phase	Size (nm)	Application
NbCl_5_ in HCl [31]	24 and 210	Nanorods	Monoclinic after being calcined at 450 °C	D: 22 L: 230	Lithium-ion batteries and dye-sensitized solar cells
Ammonium niobate oxalate hydrate, sucrose and HCl in deionized water [32]	12 and 180	Nanocomposites	Pseudo-hexagonal	D: 25–29	Highly active and stable catalyst for electrochemical reactions
Nb_2_O_5_, LiOH and NH_3_.H_2_O in H_2_O_2_ [33]	24 and 240	Hollow microspheres	Pseudo-hexagonal after being calcined at 500 °C	D: 1000–2000	Catalysis, drug carriers, and gas sensors
Ammonium niobate oxalate and hydrogen peroxide distilled water [34]	2–24 and 100–175	Nanoparticle	Orthorhombic	D: 9–35	Photoactive degradation of pollutants
Niobium foil with ammonium fluoride [35]	24–144 and 150	Nanorods	Orthorhombic for duration > 48 h	D: 50–100	Nano-scaled sensors, optoelectronic devices
Lithium hydroxide in HF acid [36]	20–40 and 150–200	Nano-trees	Pseudo-hexagonal	D: 30–500	UV sensors
Ammonium niobate oxalate hydrate and oleic acid in triethylamine [37]	2–6 and 180	Nanorods	Pseudo-hexagonal	D: 5–20 L: 100–500	Photoactive degradation of pollutants
Ammonium niobate oxalate hydrate in distilled water [37]	1 and 580	Nanospheres	Pseudo-hexagonal	D: 20–50	Photoactive degradation of pollutants
Niobium ethoxide, diethylene glycol and acetone in water [38]	4–12 and 180	Mesoporous spheres	Pseudo-hexagonal	D: 400–500	Highly effective solid acid catalysts
NbCl_5_ in ethanol mixed with triblock copolymer dissolved in distilled water [39]	24 and 110	Mesoporous	Orthorhombic after being calcined at 600 °C	N/A	N/A
NbO_2_ powder in distilled water and ethanol containing 1 M urea [40]	72–720 and 130	Nanosheets	Orthorhombic and monoclinic	T: 3–5	High reversible charge/discharge capacity and cycling stability
Nb powder in distilled water [41]	72–720 and 200	Nanorods	Orthorhombic	D: 50	An efficient material synthesized without catalyst
NbCl_5_ and ethanol in cyclohexanol [42]	8–90 and 200–240	Nanocables and nanorods	Orthorhombic	D: 50–80 L: >1000	High-performance optoelectrical devices
Nb powder in urea [43]	24–336 and 170–200	Nanobelts	N/A	W: ~60 T ~15	Lithium-ion batteries and dye-sensitized solar cells
Niobium Penta butoxide in toluene [44]	2 and 300	Nano powders	Pseudo-hexagonal	L: <80	Photocatalytic dehydro-genation of methanol in an aqueous solution under deaerated conditions
NbCl_5_ in anhydrous benzyl alcohol [45]	72 and 250	Nanoparticles	Pseudo-hexagonal	D: 18–35	High-rate-performance supercapacitor
NbCl_5_ and ethanol in cyclohexanol [46]	8–90 and 200–240	Nanograins and nanoparticles	Orthorhombic for T > 225 °C	D: 50–80 L: >1000	Catalysis and its structure–activity relationships
Oxalic acid and hydrogen peroxide [47]	4–12 and 150–220	Nanorods and nanospheres	Octahedral	L: 5	Used as nano catalyst
Oxalic acid and hydrogen peroxide [48]	4–12 and 150–220	Nanospheres and mesoporous	Monoclinic	D: 6	Used as nano catalyst
Aluminum niobium oxalate with deionized water and ethanol [49]	24 and 180	Nanorods and nanospheres	Orthorhombic after being calcined at 600 °C	D: 300–500 D: 5–10 L: 100	Higher photocatalytic activities (organic pollutants, dye sensitized and solar cells)
Nb_2_O_5_ with isopropanol [50]	48 and 180	Nanobelts	Pseudo-hexagonal	D: 100–200	Energy storage electrodes
Nb powder with distilled water [51]	72 and 180	Flake, Nanorods, Springs	Polycrystalline	L: 20–100	Nano-scaled sensor, solar cells
Ammonium oxalate with Nb_2_AlC [52]	21 and 180	Nanorods	Hexagonal and orthorhombic	D: 20–50 L: 70–150	Catalyst and water treatments
Nb with distilled water [53]	72 and 150	Nanoflakes	Orthorhombic	D: 46–53	High-performance optoelectrical devices
Nb_2_AlC with Hydrofluoric acid [54]	20 and 190	Nanorods	Monoclinic	L: 60–180	Photocatalyst
Niobium with distilled water [29]	20–40 and 130–150	Nanorods	Octahedral	D: 20–50	Biomedicine and environmental monitoring

**Table 3:** A summary of Nb_2_O_5_ properties and applications versus other commonly used nanoparticles.

Property	Niobium Pentoxide Nanoparticles (Nb_2_O_5_NPs) [11]	Gold Nanoparticles (AuNPs) [66,67]	Silica Nanoparticles (SiO_2_NPs) [68]	Silver Nanoparticles (AgNPs) [69]	Zinc Oxide Nanoparticles (ZnONPs) [70]	Carbon-Based Nanoparticles [69,71]	Titanium Dioxide Nanoparticles (TiO_2_) [68,72]
Chemical Composition	Niobium and oxygen	Pure gold	Silicon dioxide	Pure silver	Zinc and oxygen	Carbon (e.g., fullerenes, graphene)	Titanium and oxygen
Size Range (nm)	10–200 nm	1–100 nm	5–500 nm	1–100 nm	10–200 nm	Varies (e.g., graphene sheets, carbon dots)	1–100 nm
Morphology	Crystalline or amorphous	Spherical, rods, others	Spherical, mesoporous, others	Spherical, triangular, others	Spherical, rod-shaped, others	Spherical, tubular (e.g., nanotubes), sheets	Spherical, rods, others
Surface Modification	Functionalized with organic groups, metals, or polymers	Thiol groups, PEGylation, antibodies	Functionalized with silanes, organic groups, or biomolecules	Citrate, PEGylation, antibodies	Organic molecules, polymers	Functionalized with various chemical groups	Coatings, doping with metals or non-metals
Optical Properties	High refractive index, photoluminescent	Surface plasmon resonance (SPR)	Transparent, adjustable refractive index	Strong SPR, antimicrobial	UV absorption, photoluminescent	Fluorescence (e.g., carbon dots), high surface area	UV absorption, photo catalytic activity
Electrical Properties	High dielectric constant, resistive switching	Conductive, plasmonic effects	Insulator	Conductive	Semi-conductor	Conductive (graphene), semi-conducting (carbon dots)	Semi-conductor
Thermal Stability	High	Moderate	High	Moderate	High	High	High
Biocompatibility	Moderate; dependent on surface functionalization	High; excellent for biological applications	High; generally inert	Variable; size- and concentration-dependent	Generally good; antimicrobial properties	High (e.g., graphene oxide), variable for others	Generally good; dependent on surface properties
Toxicity	Low; surface-dependent	Low; size- and concentration-dependent	Generally low	Potential cytotoxicity; dose-dependent	Low; antimicrobial properties	Low; dependent on form and functionalization	Low; dependent on crystal structure and surface properties
Key Applications	Catalysis, electrochromic devices, gas sensors, photocatalysis, batteries	Biomedical imaging, drug delivery, photothermal therapy, sensors	Drug delivery, imaging, coatings, catalysis, chromatography	Antimicrobial agents, biosensing, medical imaging	Sunscreens, antibacterial agents, photocatalysis, sensors	Drug delivery, imaging, electronics (e.g., graphene)	Photocatalysis, UV blockers, sensors, biomedical applications
Unique Features	High photocatalytic activity, ion storage capabilities, high refractive index	Strong SPR for optical applications, high specificity with functionalization	High surface area (e.g., mesoporous), high chemical stability	Potent antimicrobial activity, strong SPR	Antimicrobial, UV-blocking properties, semi-conductor behavior	Exceptional mechanical strength (graphene), tunable electronic properties	Photocatalytic efficiency, UV absorption, chemical stability
Synthesis Methods	Sol–gel, hydro- thermal, precipitation	Chemical reduction, seed-mediated growth	Stöber process, sol–gel methods	Chemical reduction, photo-chemical methods	Sol–gel, hydro-thermal, precipitation	Chemical vapor deposition (CVD), arc discharge, laser ablation	Sol–gel, hydro-thermal, chemical vapor deposition
Challenges	Limited commercial availability, complex surface functionalization	Cost of gold, stability in solutions	Aggregation in some environments, limited thermal conductivity	Potential cytotoxicity, stability under various conditions	Photocatalytic activity leading to ROS generation, potential cytotoxicity	Production scalability (e.g., graphene), potential cytotoxicity	Control over crystal phase, potential environmental impact
Cost	Moderate	High	Low to moderate	Moderate	Low	Variable (e.g., graphene can be expensive)	Low
